# Forensic psychiatry in Pakistan: Where next following the Supreme Court judgement

**DOI:** 10.1192/j.eurpsy.2022.1536

**Published:** 2022-09-01

**Authors:** T. Hassan

**Affiliations:** Providence Care Hospital, Psychiatry, Kingston, Canada

**Keywords:** forensic, pakistan, psychiatry, justice

## Abstract

**Introduction:**

No statutory mental health services exist for justice-involved individuals in Pakistan. The lack of expertise in forensic psychiatry serves to deny individuals with mental illness the critical support needed for mental healthcare and adequate court dispositions with serious unintended consequences including capital punishment for those who could otherwise be deemed treatment and not punishment worthy. A landmark judgement by the Supreme Court of Pakistan in February 2021 criticized the lack of forensic psychiatry expertise in Pakistan and directing the development of forensic mental health services and forensic psychiatry training in Pakistan.

**Objectives:**

The key objectives are: 1. Understanding the timeline of how justice invloved individuals are manged by psychiatric services 2. The importance of the Supreme Court of Pakistan Judgement in affecting change 3. Highlights on how Queen’s University will enhance forensic psychiatry in Pakistan

**Methods:**

A literature review and personal networking facilitated the collection of important data in how justice invloved individuals are supported in Pakistan. The author has published and presented to Pakistani psychiatrists and the Pakistani judiciary on this topic. Queen’s University is aiming to implement a 3-year plan to develop an online curriculum and certificate course to help train the trainers.

**Results:**

In the Pakistan’s most populous province, Punjab, prevalence rates for psychotic illnesses (3.7%), major depression (10%), and personality disorders (65%) among men with higher rates for psychotic disorders (4.0%) and major depression (12%) among women.

**Conclusions:**

In conclusion there is a dire need to develop forensic psychiatry in Pakistan and other low/middle income countries.

**Disclosure:**

No significant relationships.

